# Prognostic and clinicopathological roles of circular RNA expression in chemoresistance in head and neck squamous cell carcinoma: a systematic review

**DOI:** 10.3389/fphar.2025.1502107

**Published:** 2025-03-19

**Authors:** Sayan Kumar Das, Sameer Khasbage, Ashim Mishra, Babban Jee

**Affiliations:** ^1^ Department of Pharmacology, Manipal Tata Medical College, Manipal Academy of Higher Education, Manipal, India; ^2^ Department of Pharmacology, People’s College of Medical Sciences and Research, Bhopal, India; ^3^ Department of Forensic Medicine, Manipal Tata Medical College, Manipal Academy of Higher Education, Manipal, India; ^4^ Department of Research, Manipal Tata Medical College, Manipal Academy of Higher Education, Manipal, India

**Keywords:** circular RNA, microRNA, biomarker, chemoresistance, head and neck squamous cell carcinoma, systematic review

## Abstract

**Background:**

Characterized by a poor prognosis and survivability, head and neck squamous cell carcinoma (HNSCC) is an aggressive neoplastic condition with a propensity for recurrence where the development of chemoresistance adversely affects the prognostic outcome. Recently, it was shown that circular RNAs (circRNAs) augment the cellular survivability and chemoresistance of malignant cells. Hence, biomarkers for early detection of chemoresistance in these patients can significantly aid in preventing a poor prognostic outcome.

**Objective:**

The present study aimed to systematically identify circRNAs that play a vital role in the development of chemoresistance in HNSCC and understand their mechanisms of action in HNSCC chemoresistance.

**Methods:**

The protocol was prospectively registered on PROSPERO with protocol no. CRD42024532291. A six-stage methodological and PRISMA recommendations were followed for the review.

**Results and Discussion:**

13 studies were identified which yielded 13 circRNAs which have been investigated for their role in the chemoresistance in HNSCC. Of these, 11 circRNAs were reported to be upregulated while only 2 circRNAs were found to be downregulated. Moreover, we found that circRNAs can modulate autophagy (circPARD3, circPKD2, circAP1M2 and circPGAM1), apoptosis (circ-ILF2, circANKS1B, circTPST2, circPUM1 and circ_0001971), drug efflux (circ-ILF2, has_circ_0005033 and circTPST2), EMT (circANKS1B, circCRIM1, circ_0001971), tumor microenvironment (circ-ILF2. circ-ILF2, circCRIM1 and circTPST2), DNA damage (circTPST2) and malignant potential (hsa_circ_0000190 and hg19_ circ_0005033).

**Conclusion:**

The present study identified 13 circRNAs which may serve as biomarkers for prognosis as well as response to chemotherapy in HNSCC.

**Systematic Review Registration:**

PROSPERO, identifier CRD42024532291.

## Graphical Abstract

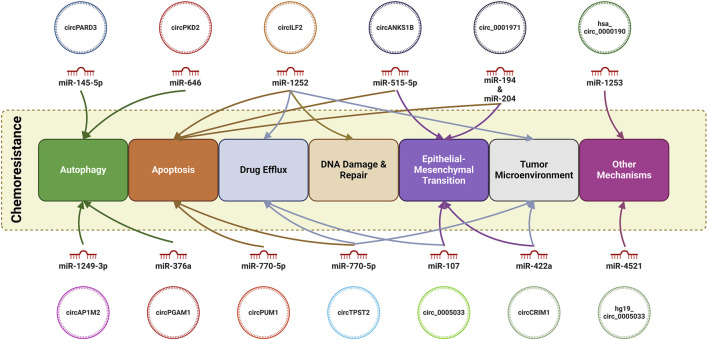

## 1 Introduction

Malignancies arising from the squamous cell lining of the tissues of the oral cavity, nasal cavity, pharynx, larynx, lip, paranasal sinuses and salivary glands, collectively are termed as head and neck squamous cell carcinoma (HNSCC). With a prevalence of an estimated 890,000 new cases and 450,000 deaths per year, HNSCC poses a serious health problem in especially in South and Southeast Asian countries and accounts for 4.5% of cancer diagnosis globally. With an annual incidence of about 380,000 cases, oral squamous cell carcinomas (OSCCs) are the most common form of HNSCC, followed by laryngeal squamous cell carcinoma (LSCC), nasopharyngeal carcinoma, and cancers of the hypopharynx and salivary glands (Barsouk et al., 2023). Risk factors for the development of HNSCC can be both non-infectious such as consumption of tobacco with or without areca nut and alcohol, exposure to carcinogenic environmental pollutants including organic, inorganic chemicals, particulate matter (PM), poor oral hygiene, malnutrition/diet lacking fruit and green vegetables, ageing or infectious such as human papillomavirus (HPV) for oropharyngeal carcinoma and Epstein-Barr virus (EBV) for nasopharyngeal carcinoma (Johnson et al., 2020).

Depending on the primary tumor location and TNM staging, the treatment options entail surgery, radiation and chemotherapy in various combinations. In 40% of the patients, presenting at an early stage (Stage I and II) of the disease, surgery is the primary modality of treatment whereas for patients with advanced disease (Stage II and IV), irrespective of the resectability of the tumor, the palliative treatment involves platinum-based chemotherapy and radiation, with or without induction chemotherapy (Marur and Forastiere, 2016). The addition of targeted therapy along with cytotoxic chemotherapy although has resulted in the prolongation of the median survival time to 10 months, this is indicative of the lack of chemotherapeutic efficacy for these patients (Sacco and Cohen, 2015). The development of chemoresistance in patients of HNSCC remains a major cause of concern as it worsens the prognostic outcome. A plethora of mechanisms for development of chemoresistance in HNSCC have been postulated which includes aberrant modulation of pathways regulating apoptosis, DNA damage and repair, epithelial mesenchymal transition (EMT), modulation of cell cycle phases, cancer stem cells, epigenetic modulation, non-coding RNA (ncRNA) processing, regulation of autophagy and immune cell interactions (Khera et al., 2023). Stemming from a poor understanding of the molecular mechanism(s) of malignancy, patients of HNSCC are subjected to the same standardized regimen irrespective of their genetic variability. Hence, investigation for the elicitation of molecular biomarkers of HNSCC will not only aid in the stratification of the patients by personalizing the therapeutic approaches but also aid in minimizing toxicity and predicting the prognostic and therapeutic outcome (Khera et al., 2023).

Circular RNAs (circRNAs) are covalently closed circular molecules of endogenous RNA which are refractory to the actions of RNA exonuclease due to the absence of 5′ or 3′ termini, thus, making them more stable than linear RNA molecules such as microRNAs (miRNAs) or long non-coding RNAs (lncRNAs) and this feature makes them ideal candidates for use as biomarkers (Liu and Chen, 2022). CircRNAs have been implicated as key regulators of various physiological and pathological conditions, including malignancies and their expression is tissue and cell specific (Hwang and Kim, 2024). Acting primarily as competing endogenous RNAs (ceRNAs), circRNAs, by sponging of miRNAs, regulate the expression of downstream miRNA target genes by alleviating the inhibitory effects of the miRNA. Additionally, some circRNA, by binding to RNA-binding proteins or translating into proteins can modulate biological functions (Bach et al., 2019). In cancers, circRNAs have been observed to induce the development of chemoresistance by augmenting the efflux of drugs, regulation of apoptosis, modulation of the tumor microenvironment, autophagy and dysregulation of DNA repair (Liu et al., 2022).

In this review, we aim to systematically identify circRNAs played regulatory role in chemoresistance and explore their underlying mechanism(s) in HNSCC for application as predictive biomarkers for prognosis and response to chemotherapy.

## 2 Methods

### 2.1 Protocol and registration

The present systematic review was done as per the “PRISMA (Preferred Reporting Items for Systematic Reviews and Meta-Analyses)” statement (PRISMA statement, 2024). The protocol was prospectively registered with PROSPERO (International Prospective Register of Systematic Reviews) (Protocol No. CRD42024532291).

### 2.2 Criteria for study selection

Randomized and non-randomized controlled trials, observational studies, animal studies, *in vitro* studies were considered for the inclusion whereas systematic reviews (with or without meta-analysis), narrative reviews, editorials, letters to the editor, conference proceedings and abstracts and *in silico* studies were excluded. Studies which were published in English language or could be translated to English language were considered for the review.

### 2.3 Search strategy and study selection

The search strategy was created after reviewing published literature and discussion among the coauthors. An electronic literature search was performed in PubMed, Embase, Web of Science and Cochrane Library using predetermined keywords and MeSH terms. A bibliographic search of the articles included in the review was also performed. The detailed search strategy was mentioned in the Supplementary Material (Supplementary Table S1).

### 2.4 Data extraction

**FIGURE 1:**
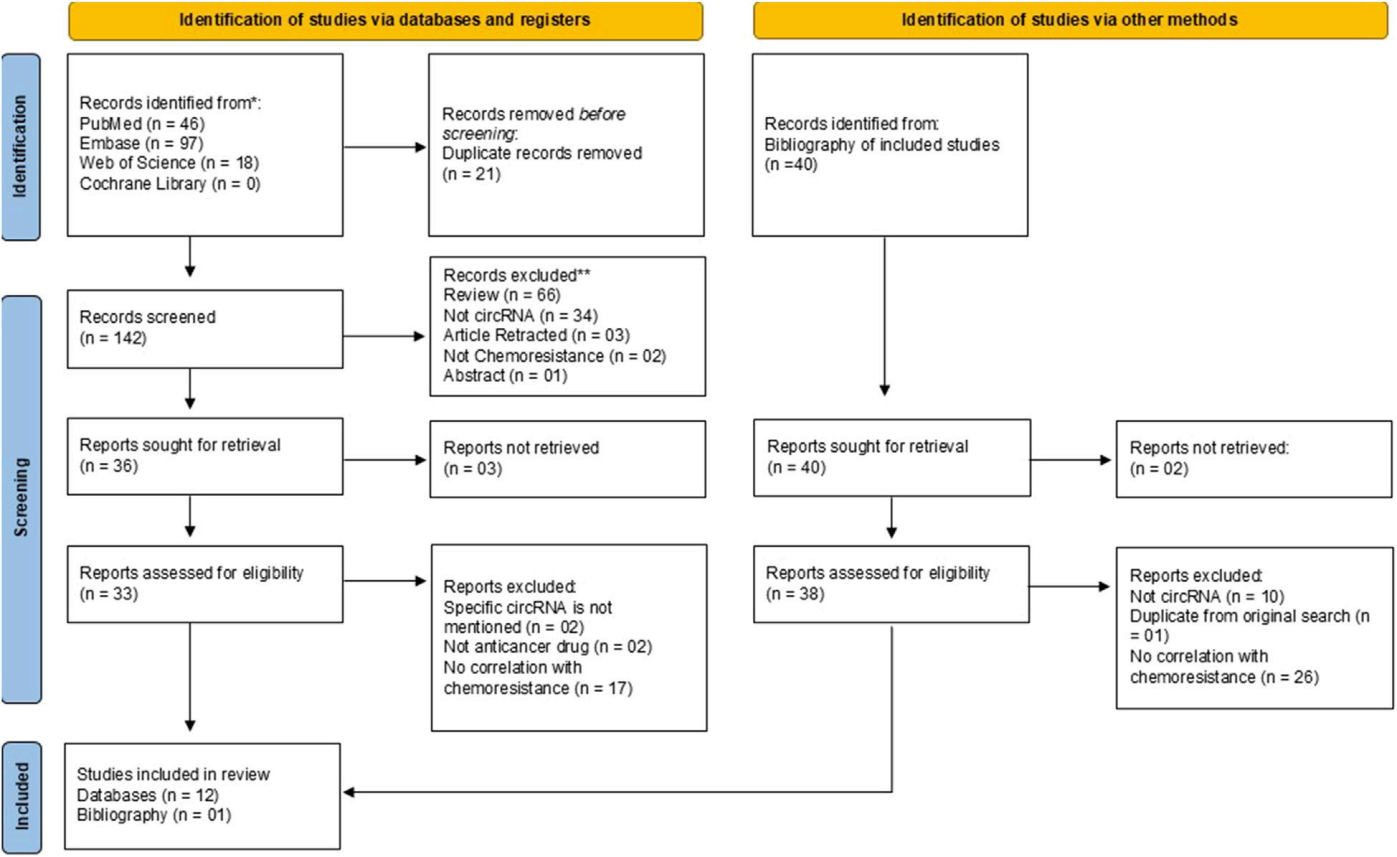
PRISMA flow diagram.

The search of four databases yielded a total of 161 articles on 27 March 2024. Of 161 articles, 46 articles were retrieved from PubMed, 97 articles from Embase, 18 articles from Web of Science and zero articles from Cochrane Library (Figure 1). All articles were exported to Microsoft Excel. Duplicates were removed using the Title-Author-Date of Publication criteria. In the first phase, title/abstract screening was performed by two individual reviewers (AM and SK) to remove 19 duplicates. The remaining 142 articles were screened for each step of the screening process in the second phase (SKD and SK) and full text screening for the assessment of eligibility for inclusion was performed by two independent authors (SKD and BJ). The bibliography of the articles included for review was also screened for identification of eligible articles for inclusion by two authors (AM and BJ). Two authors (SKD and SK) independently extracted the data using a pre-structured form where the details were recorded as: author, year, country, type of HNSCC, circRNA, circRNA regulation, target miRNA and other downstream effectors. Any discrepancy, arising at any stage of the of the review was addressed by discussion among the authors till a consensus was reached.

### 2.5 Study outcome

This study aimed primarily to evaluate the role of circRNAs in chemoresistance in HNSCC as well as to assess their prognostic potential in HNSCC cases.

### 2.6 Quality assessment of studies

Two authors (SKD and SK) assessed the risk of bias and applicability concerns independently using QUADAS-2 tool (A tool for the quality assessment for diagnostic accuracy studies) (QUADAS-2, 2024). The studies were assessed for Risk of Bias and Applicability Concerns. The risk of bias dimension had four domains whereas the dimension of applicability concerns had three domains. For risk of bias assessment, each domain was graded as “yes,” “no” and “unclear” whereas each domain for applicability concern was graded as “high” “low” or “unclear.”

### 2.7 Data synthesis and summary measures

The data extracted were categorized, interpreted, and grouped according to the similarity of the data for the narrative synthesis.

## 3 Results

### 3.1 Study selection

Following the search, a total of 161 records from four databases were obtained. After the removal of duplicates, there were 142 records screened for eligibility, out of which 36 records were sought for retrieval. A total of 12 studies from three databases were included for the review. From the bibliography of the included 12 studies, 40 studies were identified and sought for retrieval, out of which 38 were assessed for inclusion into the review. Of 38, only 1 study was found eligible. The PRISMA flow diagram (Figure 1) outlines the process of selections for the included studies.

### 3.2 Risk of bias and applicability concerns within studies

**FIGURE 2:**
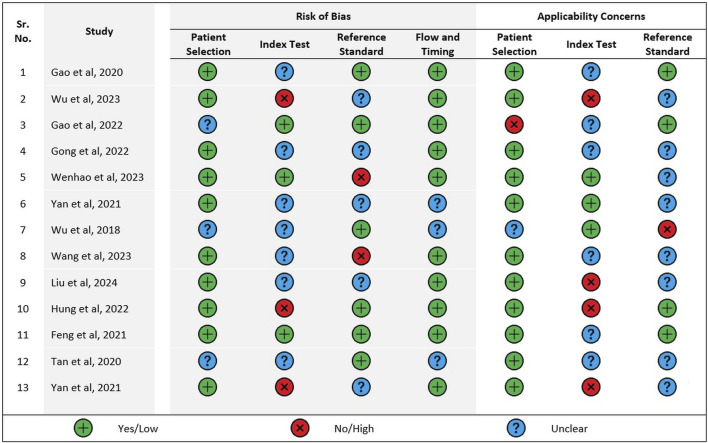
Quality assessment of included studies using QUADAS-2.

A summary of the quality assessment of the included studies using QUADAS-2 has been given in Figure 2. Only one study fulfilled all the domains as mentioned under risk of bias in the QUADAS-2 quality assessment tool whereas most studies satisfied only 2 domains. Only four studies satisfied 2 domains pertaining to applicability concerns and none satisfied all three domains.

### 3.3 Study characteristics

The characteristics of studies included in the review have been summarized in Table 1.

**TABLE 1:** Characteristics of included studies.

No	Author (Year)	Country	Type of HNSCC	circRNA	circRNA regulation	Chemotherapeutic agent	Target miRNA	Other downstream targets
1	Gao et al. (2020)	China	Laryngeal squamous cell carcinoma (LSCC)	circPARD3	Upregulated	Cisplatin	miR-145-5p	PRKCI
2	Wu et al. (2023)	China	Oral squamous cell carcinoma (OSCC)	circ-ILF2 (hsa_circ_001428)	Upregulated	Cisplatin	miR-1252	KLF8
3	Gao et al. (2022)	China	Oral squamous cell carcinoma (OSCC)	circ-PKD2	Downregulated	Cisplatin	miR-646	Atg13
4	Gao et al. (2022)	China	Laryngeal squamous cell carcinoma (LSCC)	hsa_circ_0005033	Upregulated	Cisplatin	miR-107	IGF1R
5	Wenhao et al. (2023)	China	Oral squamous cell carcinoma (OSCC)	circAP1M2	Upregulated	Cisplatin	miR-1249-3p	ATG9A
6	Yan and Xu (2021)	China	Oral squamous cell carcinoma (OSCC	circANKS1B	Upregulated	Cisplatin	miR-515-5p	TGF- β1
7	Wu et al. (2018a)	China	Laryngeal squamous cell carcinoma (LSCC)	hg19_circ_0005033	Upregulated	Cisplatin	miR-4521	-
8	Wang et al. (2023)	China	HNSCC subtype not specified	circTPST2	Upregulated	Cisplatin	miR-770-5p	Nucleolin
9	Liu et al. (2024)	China	Oral squamous cell carcinoma (OSCC)	circPUM1	Upregulated	Cisplatin	miR-770-5p	NK cells & NAP1L1
10	Hung et al. (2022)	Taiwan	Oral squamous cell carcinoma (OSCC)	hsa_circ_0000190	Downregulated	Cisplatin	-	-
11	Feng et al. (2021)	China	Laryngeal squamous cell carcinoma (LSCC)	circPGAM1	Upregulated	Cisplatin	miR-376a	ATG2A
12	Tan et al. (2020)	China	Oral squamous cell carcinoma (OSCC)	circ_0001971	Upregulated	Cisplatin	miR-194 & miR-204	-
13	Hong et al. (2020)	China	Nasopharyngeal carcinoma	circCRIM1	Upregulated	Docetaxel	miR-422a	FOXQ1

### 3.4 Differentially expressed circRNAs

#### 3.4.1 circPARD3

Gao et al., in their study, identified a novel autophagy-suppressive cytoplasmic circRNA, i.e., circPARD3, which was significantly upregulated in LSCC samples. miR-145-5p was identified as a downstream target for circPARD3, where the sponging of miR-145-5p by circPARD3 alleviated its inhibitory effects resulting in the overexpression of oncogene PRKCI. circPARD3 was found to inhibit autophagy via the modulation of the miR-145-5p-PRKCI-Akt-mTOR pathway, resulting in augmentation of the neoplastic potential of LSCC and precipitation of chemoresistance. The level of expression of circPARD3 is LSCC was seen to be directly corelated with the malignant progression and worsening of the prognostic outcome (Gao et al., 2020).

#### 3.4.2 circPKD2

Gao and his colleagues observed that circPKD2 was significantly downregulated in OSCC cells. Further, they found that circPKD2 promotes autophagy by sponging of miR-646 which in turn negated its suppressive effects on Atg13. The decreased expression of circ-PKD3 in OSCC cells was associated with tumor proliferation, invasion and chemoresistance (Gao et al., 2022).

#### 3.4.3 circAP1M2 (hsa_circ_0049282)

In the study conducted by Wenhao and his group, an increased expression of circAP1M2 in OSCC tissue was observed. In comparison to parental cells, circAP1M2 was noted to be significantly overexpressed in cisplatin-resistant OSCC cells. circAP1M2 was found to regulate autophagy by sponging of miR-1249-3p which in turn modulates the expression of ATG9A (Wenhao et al., 2023).

#### 3.4.4 circPGAM1

Feng et al. observed the upregulation of circPGAM1 in laryngocarcinoma tumors where the level of expression of circPGAM1 showed a positive correlation with the higher stages (Stages III and IV) of malignancy in comparison to the lower stages (Stages I and II). circPGAM1 was seen to promote chemoresistance as its downregulation was associated with a decrease in cell viability in the presence of cisplatin. MiR-376a was identified as the downstream target of circPGAM. MiR-376a inhibited the circPGAM1 induced increase in cell viability under cisplatin. ATG2A was identified as a downstream factor for miR-376a where the miR-mediated inhibition of ATG2A promoted cisplatin sensitivity (Feng et al., 2021).

#### 3.4.5 circ-ILF2 (hsa_circ_00428)

Wu et al. identified upregulated circ-ILF2 (hsa_circ_00428) in CDDP-resistant OSCC cells. miR-1252 was identified as a target for circ-ILF2, where sponging of miR-1252 inhibited the latter’s effects on KLF8 expression, resulting in the development of cisplatin resistance in OSCC cells because of impairment of cisplatin-induced apoptotic machinery. circ-ILF2 was also found to promote the M2 polarization of macrophages (Wu et al., 2023).

#### 3.4.6 circ_0005033 (hsa_circ-0005033)

In a study, Gong and co-workers observed that circ_0005033 was upregulated in LSCC specimens. miR-107 was identified as the target for circ_0005033 and the relationship was seen to be an inverse linear correlation. The upregulation of circ_0005033 and inhibition of miR107 were associated with augmentation of LSCC cell viability, proliferation, metastasis and chemoresistance to cisplatin. The downstream effector for miR107 was identified to be IGF1R (Gong et al., 2022).

#### 3.4.7 circANKS1B (hsa_circ_0007294)

In a study, we demonstrated the elevated levels of circANKS1B and TGF-β1 in OSCC tissues in comparison to the para-tumor samples and suggested a positive correlation where circANKS1B was found to regulate the expression of TGF-β1 in OSCC. circANKS1B upregulation was associated with an elevation of the metastatic potential of the OSCC cells. Downregulation of circANKS1B resulted in decreased cell viability when exposed to cisplatin and an increase in the activity of caspase-3. The expression of miR-515-5p was seen to be noticeably downregulated in OSCC and a direct interaction with circANKS1B was noted suggesting of miRNA sponging. miR-515-5p was observed to reduce the mRNA levels of TGF- β1 suggestive of a circANKS1B/miR-515-5p/TGF- β1 axis (Yan and Xu, 2021).

#### 3.4.8 hg19_circ_0005033

Wu et al., in their study, observed high level expression of hg19_circ_0005033 in TDP cells of LSCC. siRNA transfection induced downregulation of hg19_circ_0005033 which inhibited proliferation, migration and invasion of TDP cells and an enhancement of chemosensitivity. An inverse relationship between hg19_circ_0005033 and miR-4521 was observed which suggests that hg19_circ_0005033 exerts its action via the sponging of miR-4521. STAT5A was identified as the target for miR4521 and downregulation of miR-4521, due to the sponging by hg19_circ_0005033, upregulated the expression of hg19_circ_0005033 via the hg19_circ_0005033/miR-4521/STAT5A axis (Wu Q. et al., 2018).

#### 3.4.9 circTPST2

Wang et al. identified circTPST2 to be relatively highly expressed in HNSCC in comparison to the adjacent tissue. miR-770-5p was identified as the downstream target for circTPSP2. Further, physical interaction between circPTST2 and nucleolin and regulation of nucleolin by miR-770-5p were established. We found that circTPSP2 regulates cisplatin chemosensitivity by sponging miR-770-5p which in turn modulated the Nucleolin dual pathway (Wang et al., 2023).

#### 3.4.10 circPUM1

Liu et al. observed the overexpression of circPUM1 in OSCC and this overexpression was regulated by the upstream factor, SP2. An inverse relationship was observed between circPUM1 and susceptibility of NK cells where high expression of circPUM1 decreased the susceptibility of OSCC cells to NK cells. The downstream target for circPUM1 was identified to be miR-770-5p playing a role in cisplatin chemoresistance and susceptibility to NK cells of OSCC cells. Overexpression of miR-770-5p was associated with a decrease in cellular viability whereas downregulation was associated with an increase in cellular viability. Nucleosome assembly protein 1-like 1 protein (NAP1L1) was identified as a downstream target for miR-770-5p which shows an inversely correlated relationship whereas an elevated expression of miR-770-5p inhibits NAP1L1 expression. Similarly, an elevated expression of circPUM1 was found to downregulate the expression of NAP1L1 which is suggestive of an axis comprising of circPUM1/miR-770-5p/NAP1L1 (Liu et al., 2024).

#### 3.4.11 circ_0001971

Tan et al. observed significant upregulation of circ_0001971 in OSCC tumor tissues in comparison to normal tissue and the elevated expression was seen to be associated with increase in cellular proliferation, migration, invasion and EMT. The expression of circ_0001971 was significantly elevated in higher stages (Stage III and IV) of the malignancy in comparison to the lower stages (Stages I and II) and was suggestive of a relationship where the level of expression has direct correlation with the five-year survival rate. Suppression of circ_0001971 expression inhibited tumorigenesis and progression while augmenting the induction of apoptosis and chemosensitivity to cisplatin. miR-194 and miR-204 were identified as downstream targets for circ_0001971 where overexpression of the two miRNAs inhibited the expression and function of circ_0001971 suggesting that circ_0001971 functions as a ceRNA for the two miRNAs (Tan et al., 2020).

#### 3.4.12 circCRIM1 (has_circ_0002346)

Hong et al. observed overexpression of circCRIM1 in highly metastatic nasopharyngeal carcinoma (NPC) cells. circCRIM1 was found to induce migration, invasion and EMT in NPC cells. miR-422a was identified as a downstream target for circCRIM1 and was found to be downregulated. Transcription factor forkhead box Q1 (FOXQ1), an oncogene, was identified as a target for miR-422a. circCRIM1 was noted to upregulate FOXQ1 by the attenuation of the posttranscriptional suppression activity of miR-422a. circCRIM1 downregulation enhanced docetaxel sensitivity of NPC cells. Hence, circCRIM1, functioning as a ceRNA, sponges miR-422a to alleviate its inhibitory effects on FOXQ1 in nasopharyngeal carcinoma, promoting metastasis and chemoresistance (Hong et al., 2020).

#### 3.4.13 hsa_circ_0000190

Hung et al. investigated the expression of hsa_circ_0000190 and hsa_circ_0001649, which have previously been reported as potential cancer biomarkers, in plasma of OSCC patients with an aim of improving risk stratification of recurrence, metastasis and chemoresistance. Both hsa_circ_0000190 and hsa_circ_0001649 were found to be downregulated in OSCC in comparison to normal healthy individuals with the downregulation of hsa_circ_0000190 being highly statistically significant (p < 0.0001) in the late stage. hsa_circ_0000190 could effectively differentiate OSCC from normal healthy individuals. Decreased expression of hsa_circ_0001649 in OSCC patients was correlated with an increased risk of early recurrence and poor overall survival. When the pre- and post-induction of chemotherapy expression levels of both circRNAs were evaluated, a trend of increase in expression of hsa_circ_0000190 was observed which is suggestive of a correlation to induction chemotherapy in OSCC whereas no such correlation was observed with hsa_circ_0001649 (Hung et al., 2022).

## 4 Discussion

A comprehensive literature search was performed using four databases followed by a bibliographic search of included articles was performed from which a total of 13 studies investigating the role of circRNAs in the development of chemoresistance in HNSCC were included for the present review. We identified 13 cirRNAs which have a pivotal role in chemotherapy resistance in HNSCC cases. Of these 13 circRNAs, 12 circRNAs (circPARD3, circ-ILF2, cricPKD2, hsa_circ_0005033, circAP1M2, circANKS1B, hg19_circ_0005033, circTPST2, circPUM1, has_circ_0000190, circPGAM1, and circ_0001971) were investigated for drug-resistance with cisplatin whereas only 1 circRNA (circCRIM1) was investigated for Docetaxel. While most circRNAs were upregulated, only circRNA has_circ_0000190 was found to be downregulated.

Autophagy, mediated by autophagosomes, is a physiological intracellular pathway responsible for the degradation and elimination of misfolded proteins and damaged cell organelles during stressful cellular environments for maintaining cellular metabolism, energy homeostasis and promoting cellular survival (Yun and Lee, 2018). Autophagy can play a dual role in tumorigenesis as it modulates both, as a mechanism of tumor suppression or as a mechanism of cyto-protection, promoting cellular adaptation to hypoxic microenvironment which in turn facilitates chemoresistance (Yang et al., 2011). Chemotherapeutic agents like cisplatin exert their anti-neoplastic activity by the induction of autophagy, whereas modulation of autophagy can be an important aspect of enhancing chemotherapeutic sensitivity while decreasing chemoresistance specially pertaining to drug like cisplatin (Xu and Gewirtz, 2022). Hence, modulation of autophagy may aid in reducing chemoresistance and augment the sensitivity of the neoplastic cells to these chemotherapeutic agents (Pu et al., 2022). Studies demonstrated that circPARD3 inhibits autophagy by the modulation of circPARD3/miR-145-5p/PRKCI axis which in turn inhibits the PIK3CA/AKT-mTOR signaling pathway. mTOR activation via the PIK3CA/AKT-mTOR signaling pathway regulates multiple cellular processes, such as growth, proliferation, metabolism and survival which, due to constitutive activation in malignancies, drives uncontrolled cellular growth and proliferation while inhibiting apoptosis (Zheng et al., 2024). MiR-145-5p is a suppressor of tumorigenesis and an enhancer of autophagy (Gao et al., 2020). CircPARD3, by sponging miR-145-5p, downregulates its expression. The downregulation of miR-145-5p in turn suppresses the expression of PRKCI (Kim et al., 2020). First classified as a human oncogene in lung and ovarian carcinomas, PRKCI, via the inhibition of PIK3CA/AKT-mTOR signaling pathway, inhibits autophagy as reflected by a decrease in the light chain 3B-II (LC3B-II) protein, increase in p62 and weakened degradation of both endogenous and exogenous autophagic substrates (Qu et al., 2016; Inman et al., 2022). Due to the downregulation of the inhibitory effect of miR-145-5p by circPARD3, the resultant overexpression of PRKCI potentiated the migration and invasion of the LSCC cells while making them refractory to the therapeutic effects of cisplatin. Although no established PRKCI inhibitors are available for clinical use (Jiao et al., 2023), PI3K enzyme inhibitors such as buparlisib and alpelisib have the potential to improve the therapeutic outcome in patients with HNSCC (Jung et al., 2018; Razak et al., 2023). Similarly, mTOR inhibitors like everolimus have been investigated for used as an adjuvant therapy in advanced HNSCC with promising therapeutic outcomes (Nathan et al., 2022; Massarelli et al., 2015).

Cytoprotective autophagy, by the degradation of damaged organelles and preventing DNA damage, enhances cellular survival in turn promoting tumorigenesis and predisposes the development of resistance to chemotherapeutic agents (Zhang et al., 2022). Autophagosomes are double layered vesicles which form an integral component of the intracellular degradation pathway in autophagy where they deliver degraded cytoplasmic components to the lysosome for recycling (Yun and Lee, 2018). Although primarily aimed at maintaining homeostasis by facilitating the recycling of damaged cell organelles and in turn preventing tumorigenesis, autophagy under conditions of cellular stress, such as hypoxia, nutrient deprivation and chemotherapy-induced therapeutic pressure and under the influence of factors secreted by immune and stromal cells of the tumor microenvironment, aids in the recycling of cellular components which facilitates equitable distribution of nutrients and energy among the cancer cells (Chavez-Dominguez et al., 2020). The activation of autophagy-related genes, such as ATG5 and ATG7, facilitates the shift from tumor suppression to cytoprotective autophagy and hence, alteration in expression of these gene may improve the effectiveness of chemotherapeutic agents (Chavez-Dominguez et al., 2020).

CircPKD2 was observed to augment cytoprotective autophagy via the modulation of the circPKD2/miR-646/Atg13 pathway (Gao et al., 2022). Atg13 is an essential component in the early stages of autophagosome formation which, depending on its state of phosphorylation, can promote or inhibit autophagy (Bhattacharya et al., 2024). Moreover, circPKD2 was found to promote apoptosis via autophagy and in turn promoting cisplatin sensitivity by up-regulating Atg13. circPKD2 augments caspase-8 and caspase-3 cleavage (Xu and Gewirtz, 2022). Caspase-8 acts as the initiator caspase for extrinsic apoptosis (Fritsch et al., 2019) whereas caspase-3 is a part of the intrinsic apoptotic pathway and plays a role in DNA and cytoskeletal protein degradation (Brentnall et al., 2013). It was previously observed that caspase-8 activation can induce caspase-9 (Li et al., 1998), but in some studies, it was demonstrated that cisplatin-induced apoptosis was associated with the upregulation of caspase-9 activity whereas caspase-8 activity was seen to be unchanged (Park et al., 2002; Garcia-Berrocal et al., 2007). Hence, further work is warranted for examining the role of circPKD2 induced caspase-8 activity in relation to chemoresistance to cisplatin.

circAP1M2 via the regulation of miR-1249-3p modulated the expression of ATG9A (Wenhao et al., 2023). ATG9A is a multi-spanning membrane protein and plays a role in the early stages of autophagy by contributing to the expansion of the phagophore and lipid mobilization from the lipid droplets to the autophagosomes and mitochondria (Mailler et al., 2021). MiR-1249-3p has previously been implicated in augmenting the malignant potential of multiple cancers (Chen et al., 2019). The expression of miR-1249-3p was seen to be downregulated in the OSCC cells and this was primarily due to the overexpression of circAP1M2 suggesting a relationship which was negatively correlated. Sponging of the miR-1249-3p alleviated its inhibitory effects on ATG9A. This phenomenon suggested that the underlying mechanism of cisplatin resistance in OSCC may be induction of cytoprotective autophagy. Earlier it was observed that cisplatin induces both, non-protective as well as cytotoxic autophagy, which can be modulated via an “autophagic switch” stemming from the alteration of expression of specific genes (Xu and Gewirtz, 2022). Although no therapeutic modality is available for the negation of ATG, concomitant administration of mTOR inhibitors, chloroquine and hydroxychloroquine, which have been seen to play a role in the regulation of autophagy, may aid in the alleviation of chemoresistance by the modulation of this “autophagic switch” (Wang G. J. et al., 2020).

**FIGURE 3:**
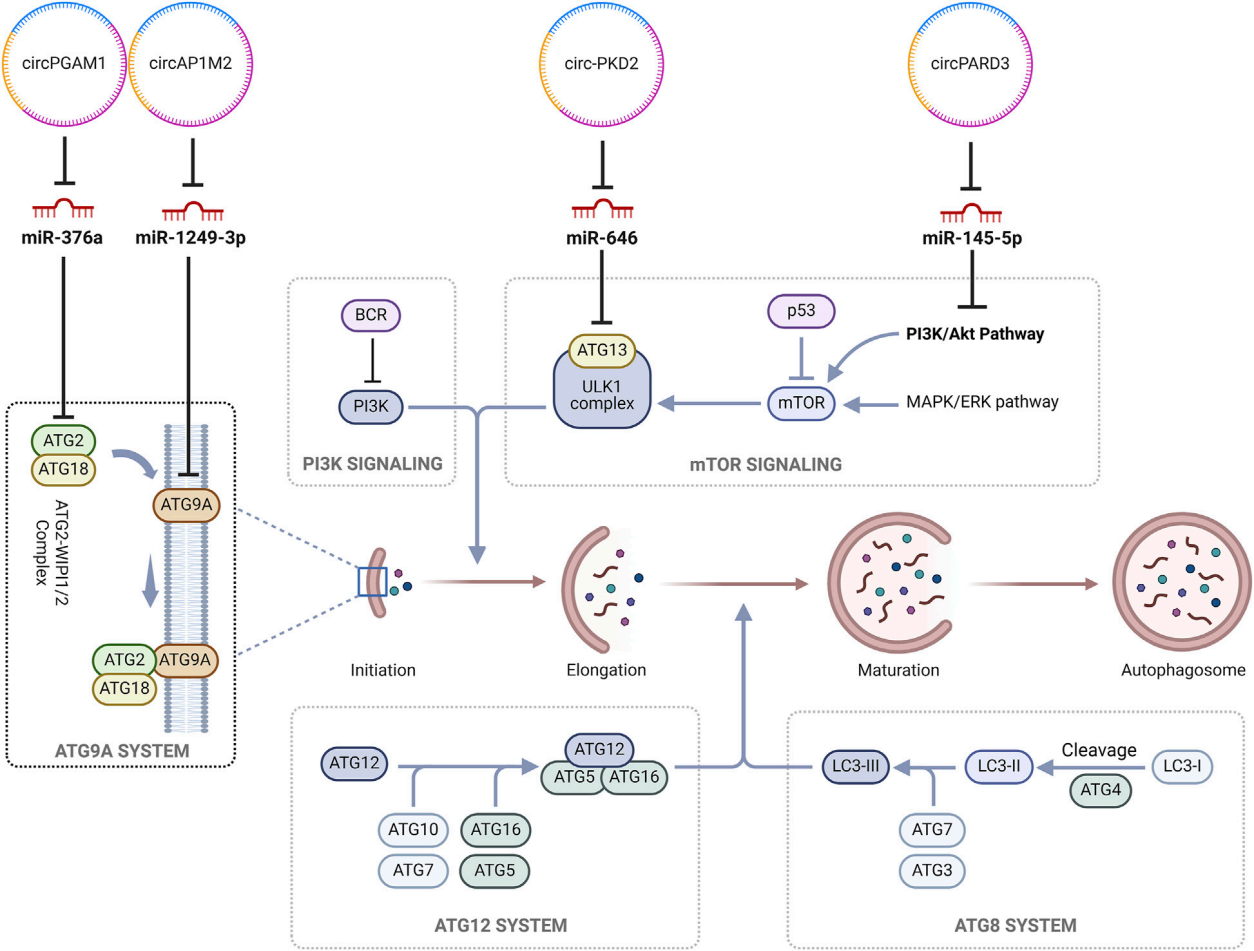
CircRNAs regulating autophagy in cancer chemoresistance. (circ, circRNAs; ATG2, Autophagy-related protein 2; ATG18, Autophagy-related protein 18; miR, MicroRNAs; BCR, breakpoint cluster region protein; PI3K, Phosphatidylinositol-3-kinase; ULK1, Unc-51-like autophagy-activating kinases 1; p53, tumor protein p53; MAPK/ERK, Mitogen-activated protein kinase/extracellular signal-regulated kinase; LC3-II, Microtubule-associated protein 1A/1B-light chain 3; mTOR, Mammalian target of rapamycin).

Similarly, circPGAM1, by sponging miR-376a, upregulated the expression of ATG2A (Feng et al., 2021). MiR-376a is a known tumor suppressor as well as a promoter of apoptosis in multiple cancers such as renal cell carcinoma (Fan et al., 2019) and osteosarcoma (Fellenberg et al., 2019), among others. In HNSCC, miR-376a has been observed to play a similar role by inhibiting ATG2A (Feng et al., 2021). ATG2A is a lipid transfer protein which plays a role in autophagosome assembly and transfer of lipids from membrane vesicles to other vesicles (Maeda et al., 2019). As a part of the ATG9/ATG12-WIPI complex, it plays a role in the recruitment of ATG9 for the expansion of autophagosomes (Li et al., 2020). The downregulation of ATG2A has been observed to induce an accumulation of immature autophagosome membranes which in turn promote the activation of non-canonical caspase-8 via an intracellular death-inducing signaling complex (DISC) (Tang et al., 2017). Hence, circPAGM1 mediated upregulation of ATG2A resulted in the induction of cytoprotective autophagy which precipitates cisplatin chemoresistance. Figure 3 delineates the roles of the identified circRNA in the autophagy pathway in cancer chemoresistance.

An important mechanism of development of chemoresistance is an increase in the efflux of cytotoxic drugs (Kannampuzha and Gopalakrishnan, 2023). The upregulation of circILF2 (hsa_circ_00428) in OSCC, by sponging miR-1252, alleviated its inhibitory effects on KFL8 which in turn preventing cisplatin-induced apoptosis (Wu et al., 2023). As a regulator of gene transcription, Krüppel-like factor 8 (KFL8), plays a vital role in multiple cellular process such as differentiation, apoptosis, drug resistance and inflammation, and has been implicated in the progression of various neoplastic conditions including triple negative breast cancer (TNBC), lung adenocarcinoma, hepatocellular carcinoma, gastric carcinoma and osteosarcoma among others (Liu et al., 2017; Le Minh et al., 2023; Kumar et al., 2021). KLF8 has been observed to promote chemoresistance via PARP-1 dependent DNA damage response in HCC cells. In solid tumors, KLF8, on binding to the upstream region of the MDR1, promotes translational activity in the tumour microenvironment under hypoxic conditions and thus, inhibiting apoptosis and augmenting the rate of efflux of the chemotherapeutic agent (Xu et al., 2010). M2 macrophages have been noted to induce chemoresistance and radioprotection by the secretion of growth factors and inhibition of apoptotic pathways in neoplastic cells (Jayasingam et al., 2020). Hence, circILF2 plays a role in the development of chemoresistance via two mechanisms, augmenting KLF8 expression and M2 polarization of macrophages.

circ_0005033 was upregulated in LSCC and by sponging miR-107 increased the expression of IGF1R (Gong et al., 2022). IGF1R signaling is a tightly regulated network essential for cellular proliferation and survival. Commonly overexpressed in neoplasms, this growth factor and receptor is responsible for sustained proliferative signals, inhibition of apoptosis, metastasis and chemoresistance. IGF1R induced chemoresistance includes promotion of proliferation, inhibition of apoptosis, increased expression of ABC transporter proteins and changes in the extracellular matrix (Yuan et al., 2018). Although miR-107 was found to be downregulated in LSCC, but in the hypoxic microenvironment of breast and colon cancers, it was overexpressed where it inhibits VEGF-mediated angiogenesis via the downregulation of HIF-1β-mediated signaling pathway (Bao et al., 2012). Upregulation of circ_0005033 alleviated the inhibitory effects of miR-107 on IGF1R precipitating the development of chemoresistance to cisplatin.

circANKS1B, by sponging miR-515-5p, elevated the expression of TGF-β in OSCC (Yan and Xu, 2021). Although TGF-β1 initially acts as a tumor suppressor, but later its role as a promoter of tumor growth was demonstrated. The role of TGF-β1 in the stromal-epithelial interactions and stromal fibroblast cell autonomous effects have implicated this cytokine in the regulation of tumor progression in malignancies (Bierie and Moses, 2006). The activation of alternate pathways of cellular survival or prevention of apoptosis by TGF-β1 has primarily been hypothesized for the development of chemoresistance but, it has been observed that downregulation of TGF-β1 expression too can play a role in EMT and chemoresistance. Downregulation of SMAD3 or absence of SMAD4 suppresses the expression of TGF-β1 which in turn induced the expression of anti-apoptotic proteins such as Bcl-2 and Bcl-w enhancing cell survivability to response to platinum coordinated compounds in NSCLC and 5-FU in CRC (Zhang et al., 2021). In cisplatin-resistant OSCC, TGF-β1 modulates the cancer cell stemness where it inhibited the tumor suppressor gene FOXO3a via AKT pathway, a non-canonical, SMAD-independent pathway, resulting in the increased expression of SOX2 and ABCG2, which are markers of stemness (Zhang et al., 2021).

hg19_circ_0005033 was found to be upregulated in TDP cells of LSCC and by sponging miR-4521 elevated the expression of STAT5A (Wu Q. et al., 2018). STAT5A has been implicated in various neoplastic conditions, for example, it is overexpressed in hematological malignancies and glioblastoma and suppressed in malignancies of the breast and ovaries suggesting a dual role of STAT5A as a tumor promoter or as a tumor suppressor depending on the underlying pathophysiology (Maninang et al., 2023). Further investigation is warranted to delineate the exact role of STAT5a in the augmentation of neoplastic potential of LSCC (Han et al., 2022). In mice models, the overexpression of STAT5A has been associated with the initiation of breast cancer, a contradictory observation to the tumor suppressive effects of STAT5, where the STAT5 signaling augments the expression TGF- α and in turn enhances the expression of EGFR predisposing the development of malignant changes (Halim et al., 2020). In colorectal cancer, STAT5 overexpression has been associated with a poor prognostic outcome where IL-23 signaling inhibits the expression of SOCS3, an inhibitor of STATs expression and activation. Activation of STATs downregulates p16, p21 and p27 while upregulating the expression of cyclin D1m Bcl-2 and survivin leading to the augmentation of cellular proliferation and inhibition of apoptosis. The STAT mediated overexpression of p-FAK, VEGF and MMP-2 levels and downregulation of E-cadherin expression raises the invasiveness and metastatic potential (Halim et al., 2020). Hence, therapeutic inhibition of STAT5 can aid in the restoration of chemosensitivity in these conditions. STAT5, by augmenting cellular proliferation, cancer stem cell population and EMT can predispose increased invasion and metastasis in hepatocellular carcinoma whereas in prostate cancer, it induces stem-like cell properties and EMT (Wu Y. et al., 2018; Halim et al., 2020).

As multifunctional protein playing a role in rDNA transcription, RNA metabolism and ribosome assembly, the expression of nucleolin in cancers have been hypothesized to be aberrant. Overexpression of nucleolin has been associated with the worsening of the prognostic outcome whereas the presence of cell surface nucleolin has been implicated in augmentation of the malignant potential and metastasis (Chen and Xu, 2016). circTPST2 was observed to be upregulated in HNSCC and by modulation of miR-770-5p regulated the expression of nucleolin. A physical interaction between circPTST2 and nucleolin was observed. Taken together, it is suggestive of a dual nucleolin pathway (Wang et al., 2023). MiR-770-5p has been implicated in the modulation of chemoresistance in multiple cancers like ovarian and colorectal adenocarcinomas. The upregulation of miR-770-5p, by its action as an anti-oncogene, promotes cisplatin sensitivity in ovarian cancers through downregulating the expression of NEAT1 (Zhu M. et al., 2020). In colon adenocarcinoma, miR-770-5p downregulates HIPK1 to modulate methotrexate resistance (Zhang et al., 2020). In HNSCC, circTPST2, by sponging miR-770-5p, modulates chemoresistance via upregulating nucleolin. circTPST2 was found to physically interact and induce the expression of nucleolin where the expression of nucleolin was found to be inversely corelated with the prognostic outcome of chemotherapy in HNSCC. The inhibition of nucelolin increased the number of late apoptotic cells (Wang et al., 2023). In cervical cancers, nucleolin modulates the expression of MDR1 in a YB1-dependent manner precipitating chemoresistance by augmenting drug efflux and reducing intra-cellular accumulation of the drug (Ke et al., 2021).

The expression of circPUM1 was found to be elevated in OSCC. Overexpression of circPUM1 was associated with a decrease in the susceptibility of NK cells and modulation of miR-770-5p which in turn downregulated the expression of NAP1L1 (Liu et al., 2024). The upregulation of miR-770-5p has been observed to promote EMT, invasion and metastasis in OSCC leading to an overall poor prognostic outcome (Khalilian et al., 2023). In a study carried out by Jia and colleagues, it was observed that miR-770-5p downregulates the expression of Sirt7 via the Sirt7/Smad4 signaling pathway to promote cellular migration (Jia et al., 2021). NAP1L1 overexpression has been associated with a poor prognostic outcome and has previously been found to promote cellular proliferation in various neoplastic conditions including colorectal cancer, HCC, lung adenocarcinoma, and neuroendocrine cancers (Zhu et al., 2022). Le et al. observed that NAP1L1 overexpression in HCC was associated with an augmentation of the neoplastic potential and chemoresistance to doxorubicin (Le et al., 2019). In glioma and ovarian cancers, NAP1L1, via its interaction with HDGF, activated c-Jun, an oncogenic transcription factor, to induce CCND1/CDK4/CDK6 expression resulting in cellular proliferation and chemoresistance to cisplatin (Zhu et al., 2022; Chen et al., 2021).

circRNAs function via the modulation of downstream RNA transcripts by competing with shared miRNAs (Salmena et al., 2011). circ_0001971 was highly expressed in OSCC cases and miR-194 as well as miR-204 were its downstream targets (Tan et al., 2020). miR-194 was found to inhibit cellular proliferation in OSCC by suppressing acylglycerol kinase (AGK) expression via the PI3K/AKT/FoxO3a signaling pathway acting as a tumor suppressor (Chi, 2015). AGK is an oncogene which has been observed to be overexpressed in a myriad of malignancies and plays a crucial role as a regulator of cellular growth and proliferation, invasion and metastasis and resistance to chemotherapy (Chu et al., 2021). AGK was also considered to be a promoter of resistance to paclitaxel in nasopharyngeal carcinomas and this chemoresistance properties which eventually mediate tumor growth and metastasis is facilitated by overexpressed FOXM1 via JAK2/STAT3 signaling pathway (Zhao et al., 2021; Zhu Q. et al., 2020). Depending on the underlying neoplastic condition, miR-204 has been observed to either play the role of a tumor suppressor or an oncomiR. Wu et al., in their study, observed the low expression level of miR-204 in HNSCC with JAK2 being its direct target. Attenuation of JAK2 by miR-204 in HNSCC inhibited the JAK2/JAK3 pathway preventing tumor angiogenesis and increased sensitivity to cetuximab suggesting a tumor suppressive activity (Wu Y. et al., 2018). The tumor suppressive actions and chemotherapy sensitivity enhancement activity of miR-204, via the inhibition of RAB22A, an oncogene, has been investigated for multiple cancers including nasopharyngeal carcinoma (Rajan et al., 2021; Yin et al., 2014). Further studies are warranted for identification of downstream targets for miR-194 and miR-204 in OSCC and establishment of the regulatory axis.

**FIGURE 4:**
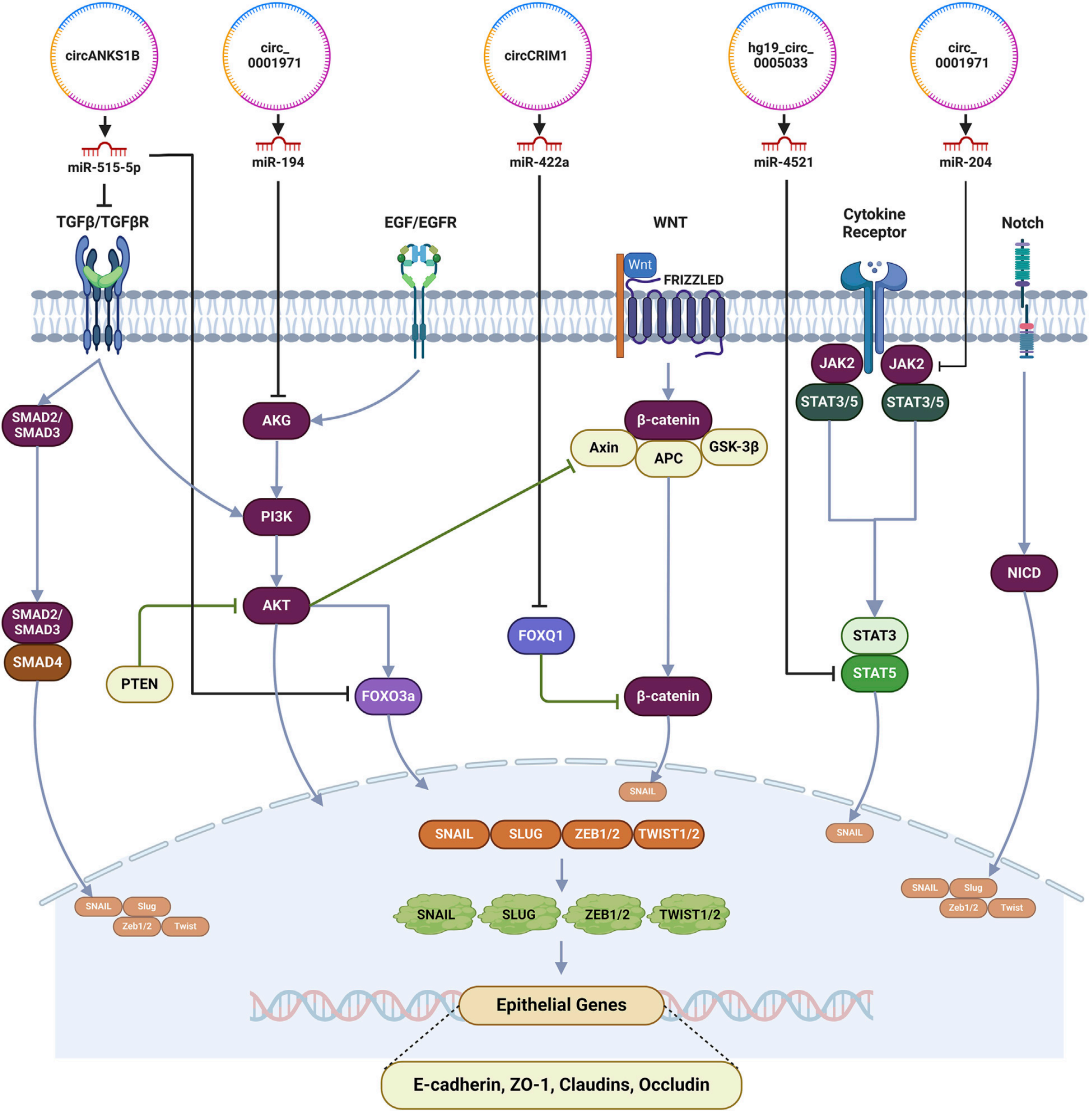
Regulation of EMT by circRNAs in HNSCC. (circ, circRNAs; ATG2, Autophagy-related protein 2; ATG18, Autophagy-related protein 18; miR, MicroRNAs; BCR, breakpoint cluster region protein; PI3K, Phosphatidylinositol-3-kinase; ULK1, Unc-51-like autophagy-activating kinases 1; p53, tumor protein p53; MAPK/ERK, Mitogen-activated protein kinase/extracellular signal-regulated kinase; LC3-II, Microtubule-associated protein 1A/1B-light chain 3; mTOR, Mammalian target of rapamycin).

circCRIM1 was observed to be upregulated in NPC cells and by sponging miR-422a alleviated the inhibitory effect on FOXQ1 in nasopharyngeal carcinoma (Hong et al., 2020). miR-422a which primarily functions as a tumor suppressor was downregulated in nasopharyngeal carcinoma (Hong et al., 2020) as well as in other neoplastic conditions like gastric carcinoma (He et al., 2018), colorectal cancer (Li et al., 2018) and osteosarcoma (Zhang et al., 2018). Moreover, miR-422a exerts its tumor suppressive effects by inhibiting the expression of FOXQ1. FOXQ1 has been implicated in the regulation of various physiological processes such as glucose metabolism, synthesis of lactic acid, cardiac fibrosis and cellular senescence. In neoplastic conditions, FOXQ1 plays a role of promoter of tumor progression and has been involved in the regulation of invasion, EMT and apoptosis (Dong et al., 2022). Zhang et al., demonstrated that FOXQ1 expression was elevated in laryngeal carcinoma and inhibition of FOXQ1 under experimental conditions impairs the cellular proliferation and invasion by arresting the cell cycle progression in G0/G1 phase (Zhang et al., 2015). Although further research is warranted to delineate the underlying mechanism(s) of FOXQ1-mediated chemoresistance in HNSCC, Meng et al. identified Twist1, Zeb2, PDGFRA and PDGFRB as potential downstream targets in breast carcinoma which are downregulated by FOXQ1 (Baumeister et al., 2021). Hence, therapeutic modalities targeting these transcription factors may play a role in the inhibition of growth, invasion and metastasis while promoting apoptosis in HNSCC. The regulation of EMT pathway by circRNA in HNSCC has been delineated in Figure 4.

Hung and colleagues investigated the clinical implications of hsa_circ_0000190 and hsa_circ_0001649 as potential biomarkers for prognosis and response to chemotherapy in OSCC. Although both circRNAs were found to be downregulated, the decreased expression of hsa_circ_0000190 in the late stage of the disease was statistically significant and it could effectively differentiate OSCC cases from normal healthy individuals. A correlation was observed between the expression of hsa_circ_0000190 and response to chemotherapy. Although the decreased expression of hsa_circ_0001649 correlated with an increased risk of early recurrence and decreased survivability, no correlation was observed between its expression and response to chemotherapy (Hung et al., 2022). Hsa_circ_0000190 has been investigated in multiple cancers where the expression varies depending on the underlying neoplastic conditions. hsa_circ_0000190 was highly expressed in non-small cell lung cancer (NSCLC) (Luo et al., 2021) while, in OSCC (Hung et al., 2022) and gastric cancer (Chen et al., 2017), it was downregulated. Although there is a paucity of data pertaining to the downstream targets and delineation of the exact role of hsa_circ_0000190 in HNSCC, a series of studies have investigated its role in the tumor progression and chemoresistance to cisplatin in NSCLC (Luo et al., 2021; Chen et al., 2017). Further, we found that hsa_circ_0000190 by sponging miR-1253 upregulated IL-6 in NSCLC to induce chemoresistance (He and Li, 2024). IL-6 has been implicated as mediator of tumor progression and chemoresistance in multiple cancers such as NSCLC and colorectal cancer wherein IL-6 has been observed to modulate autophagy by inducing phosphorylation of BECN1 via the IL-6/JAK2/BECN1 signaling pathway (He and Li, 2024; Hu et al., 2021). The phosphorylation of BECN1 at the tyrosine residue Y333 by JAK2 augments its interaction with VPS34, a component of the PI3KC3 complex, which is essential for the formation of autophagosomes in turn aiding the malignant cells to survive the stress induced by chemotherapeutic agents (Hu et al., 2021). Like in OSCC, hsa_circ_0000190 has been found to be downregulated in gastric carcinoma. miR-1252 was identified as a direct target for hsa_circ_0000190 and it directly targeted PAK3. The hsa_circ_0000190/miR-1252/PAK3 was found to modulate the malignant potential of the cells by regulating the expression of p27 and cyclin D inducing cell cycle arrest at G1 phase due to inhibition of DNA synthesis, apoptosis, cellular proliferation and migration (Wang Y. et al., 2020). As the expression of hsa_circ_0000190 varies depending on the underlying neoplastic conditions, it is a view that circRNAs exert their influence on different biological processes depending on the underlying pathology.

Similarly, hsa_circ_0001649 which is thought to be an anti-oncogenic circRNA was downregulated in multiple malignancies such as osteosarcoma (Sun and Zhu, 2020), gastric carcinoma (Li et al., 2017) and retinoblastoma (Xing et al., 2018) and is associated with a poor prognostic outcome. In osteosarcoma, it has been observed that hsa_circ_0001649, by sponging miR-338-5p, miR-647 and miR-942, inhibits the STAT signaling pathway to decrease cellular proliferation and promote apoptosis (Sun and Zhu, 2020) whereas, in retinoblastoma, hsa_circ_0001649 was observed to exert its actions via the modulation of the AKT/mTOR signaling pathway (Xing et al., 2018) and this suggests that even though dysregulated circRNA may play a role in the pathogenesis of cancer, they may not be useful as biomarkers of prognosis and response to chemotherapy.

In brief, the mechanism of development of chemoresistance in HNSCC is quite complex and involves multiple pathways regulated by a wide range of circRNAs. In present study, we found that circular RNAs circPARD3, circPKD2, circAP1M2 and circPGAM1 can modulate autophagy; circ-ILF2, circANKS1B, circTPST2, circPUM1 and circ_0001971 can regulate apoptosis and circ-ILF2, has_circ_0005033 and circTPST2 augment the efflux of the chemotherapeutic agent; EMT is enhanced by circANKS1B, circCRIM1, circ_0001971 and has_circ_0005033) whereas DNA damage and repair capacity of the cells is modulated by circ-ILF2. circ-ILF2, circCRIM1 and circTPST2 may also play a role in modulation of the tumor microenvironment. Moreover, malignant potential of cancerous cells is regulated by has_circ_0000190 and hg19_circ_0005033. Interestingly, circTPST2 circRNA is involved in regulation of apoptosis, augmentation of drug efflux, and modulation of the tumor microenvironment. Similarly, circ-ILF2 augments the drug efflux, modulates the tumor microenvironment and DNA damage.

As discussed elsewhere, the present review identifies 13 circRNAs which have been investigated for their role in chemoresistance in HNSCC. We have provided insight into their possible mechanism of action, their target miRNAs, and regulatory pathways involved in the development of chemoresistance while highlighting the potential roles of predictors of chemotherapy response in HNSCC. However, the present study has some limitations. Although we strived to conduct a comprehensive literature search involving four databases, it is possible that we may have missed literature from the grey areas. As most of the studies reported in our review have been conducted in People’s Republic of China, hence, a population bias may exist. A variation in study design including sample size, control groups and experimental designs was noted which may have contributed to some inconsistences in the results which have been reported. Majority of the studies investigated chemoresistance to cisplatin whereas only one study investigated the therapeutic response to docetaxel which may restrict the applicability to other chemotherapeutic drugs and chemotherapy regimens.

## 5 Conclusion

In this review, we have systematically identified 13 circRNAs that played a vital role in the development of chemoresistance in HNSCC. These identified circRNAs may be considered as predictive biomarkers not only for prognosis but also for response to chemotherapy. Further, these circRNAs may aid in the personalization of therapeutic modalities in HNSCC cases. The development of chemoresistance is not unidimensional but involves the modulation of multiple pathways which in turn control various cellular processes by multiple circRNAs, their target miRNAs and downstream effectors. Hence, integration of circRNAs with other biomarkers such as proteins and other ncRNAs can provide a comprehensive and more accurate understanding of the underlying pathophysiological process, treatment response and even serve as potential therapeutic targets in cancer and for deterring chemoresistance. Although all identified circRNAs should be investigated for their potential role as biomarkers, of the identified circRNAs, the authors would like to propose the four most promising candidates for further evaluation, namely, circPARD3 which modulates autophagy via the PRKCI-Akt-mTOR pathway, circTPST2 which modulates apoptosis, drug efflux and tumor microenvironment and shows a strong correlation with chemotherapy response, circANKS1B which modulation of EMT and TGF-β1 pathway (responsible for cell survival, growth and immune response) and circ_0000190 which modulates autophagy via the IL-6/JAK2/BECN1 signaling pathway and the expression of which has been reported to correlate with response to chemotherapy. Although dysregulation of expression of a circRNAs can predict the malignant potential of the cells, further exploration is required to ascertain their role in chemoresistance. Validation of the identified circRNAs and their incorporation into clinical practice warrants the conduction of longitudinal studies for observing the change in response to chemotherapy whereas *in vivo* experiments may shed light on extrapolating the role of these circRNAs in cancer at a cellular level. As discussed earlier, circRNAs are inherently more resistant to degradation than other ncRNAs owing to their closed circular structures, they hold immense potential as biomarkers, a correlation between tissue and plasma samples will aid in easier applicability. The existence of validated biomarkers with good specificity and sensitivity can aid in the early detection of chemoresistance minimizing therapy failure resulting in a better therapeutic outcome. Hence, further well-planned studies are warranted from clinical perspectives to establish the role and utility of circRNAs in chemoresistance to some other anti-cancer drugs and explore their potential as novel biomarkers for predicting prognosis and chemotherapy response.

## Data Availability

The original contributions presented in the study are included in the article/Supplementary Material, further inquiries can be directed to the corresponding author.

## Abbreviations

circRNA, Circular RNA; ceRNA, Competitive endogenous RNA; EBV, Epstein-Barr virus; EGFR, Epidermal growth factor; EMT, Epithelial mesenchymal transition; FOXQ1, Forkhead box Q1; HDGF, Hepatoma-derived growth factor; HNSCC, Head and neck squamous cell carcinoma; HPV, Human papillomavirus; IGF1R, Insulin-like growth factor 1 receptor; KFL8, Krüppel-like factor 8; LC3B-II, Light chain 3B-II; LSCC, Laryngeal squamous cell carcinoma; MMP-2, Matrix metalloproteinase-2; NAP1L1, Nucleosome assembly protein 1-like 1 protein; ncRNA, Non-coding RNA; NPC, nasopharyngeal carcinoma; NSCLC, Non-small cell lung cancer; OSCC, Oral squamous cell carcinoma; p-FAK, Phospho-focal adhesion kinase; PIK3CA/AKT-mTOR, Phosphatidylinositol 3-kinase alpha/AKT-mammalian target of rapamycin; PM, Particulate matter; TGF- α, Transforming growth factor-α; VEGF, Vascular endothelial growth factor.
