# Supplementary material for: Prognostic and clinicopathological roles of circular RNA expression in chemoresistance in head and neck squamous cell carcinoma: a systematic review
Source: Front Pharmacol. 2025 Mar 19;16:1502107. doi: 10.3389/fphar.2025.1502107 (PMC11962432; doi:10.3389/fphar.2025.1502107)
Supplement: Supplementary file 1 [file Table1.docx]

**Evaluating the prognostic and clinicopathological roles of circular RNA expression in chemoresistance in head and neck squamous cell carcinoma: A systematic review**

Sayan Kumar Das^1^, Sameer Khasbage^2^, Ashim Mishra^3^, Babban Jee^4^

^1^Department of Pharmacology, Manipal Tata Medical College, Manipal Academy of Higher Education, Manipal, India. **ORCiD:** 0000-0001-5030-4502

^2^Department of Pharmacology, People’s College of Medical Sciences and Research, Bhopal, India. **ORCiD:** 0000-0003-3235-7197

^3^Department of Forensic Medicine, Manipal Tata Medical College, Manipal Academy of Higher Education, Manipal, India. **ORCiD:** 0000-0001-6949-1627

^4^Department of Research, Manipal Tata Medical College, Manipal Academy of Higher Education, Manipal, India. **ORCiD:** 0000-0002-2530-1489

**Corresponding Author:**

Dr. Babban Jee,

Department of Research,

Manipal Tata Medical College,

Kadani Road, Baridih, Jamshedpur,

Jharkhand – 831017

*Email:* babban.jee@manipal.edu

**Supplementary Material : Table.S1**

**Search Strategy**

**Date of Search:** 27^th^ March, 2024

**Search Strategy for PubMed:**

| **#1** | "Squamous Cell Carcinoma of Head and Neck"[Mesh] | **OR** |
| --- | --- | --- |
|  | Head And Neck Squamous Cell Carcinomas |  |
|  | Squamous Cell Carcinoma of the Head and Neck |  |
|  | HNSCC |  |
|  | Squamous Cell Carcinoma of the Larynx |  |
|  | Laryngeal Squamous Cell Carcinoma |  |
|  | Squamous Cell Carcinoma of the Nasal Cavity |  |
|  | Oral Tongue Squamous Cell Carcinoma |  |
|  | Hypopharyngeal Squamous Cell Carcinoma |  |
|  | Oral Squamous Cell Carcinoma |  |
|  | Squamous Cell Carcinoma of the Mouth |  |
|  | Oropharyngeal Squamous Cell Carcinoma |  |
| **AND** | | |
| **#2** | **"Antineoplastic Agents"[Mesh]** | **OR** |
|  | antineoplastic agents |  |
|  | cancer chemotherapy |  |
|  | antitumor agents |  |
|  | antitumor drugs |  |
|  | anticancer drugs |  |
|  | anticancer agents |  |
| **AND** | | |
| **#3** | **"RNA, Circular"[Mesh]** | **OR** |
|  | circRNA |  |
|  | Closed Circular RNA |  |
|  | Circular RNA |  |
|  | Closed RNA |  |
|  | Circular Intronic RNA |  |
|  | ciRNA |  |
|  | circular exonic RNA |  |
|  | exon intron circular RNA |  |
|  | ecircRNA |  |
|  | EIciRNA |  |

**Search Strategy for EMBASE:**

| **#1** | head and neck squamous cell carcinoma [Broad search] | **OR** |
| --- | --- | --- |
|  | head and neck squamous cell carcinoma cell line [Broad search] |  |
|  | nasal squamous cell carcinoma [Broad search] |  |
|  | oral tongue squamous cell carcinoma [Broad search] |  |
|  | tongue squamous cell carcinoma cell line [Broad search] |  |
|  | hypopharynx squamous cell carcinoma [Broad search] |  |
|  | hypopharyngeal squamous cell carcinoma cell line [Broad search] |  |
|  | laryngeal squamous cell carcinoma [Broad search] |  |
|  | laryngeal squamous cell carcinoma cell line [Broad search] |  |
|  | mouth squamous cell carcinoma [Broad search] |  |
|  | mouth squamous cell carcinoma cell line [Broad search] |  |
|  | oropharynx squamous cell carcinoma cell line [Broad search] |  |
| **AND** | | |
| **#2** | antineoplastic agent [Broad search] | **OR** |
|  | cancer chemotherapy [Broad search] |  |
|  | cancer combination chemotherapy [Broad search] |  |
|  | antitumor agents [Broad search] |  |
| **AND** | | |
| **#3** | circular ribonucleic acid [Broad search] | **OR** |
|  | Circrna [Broad search] |  |
|  | closed circular rna [Broad search] |  |
|  | Cirna [Broad search] |  |
|  | circular intronic rna [Broad search] |  |
|  | circular exonic rna [Broad search] |  |
|  | exon intron circular rna [Broad search] |  |
|  | closed rna [Broad search] |  |

**Search Strategy for Web of Science:**

| **#1** | **Squamous Cell Carcinoma of Head and Neck (All Fields)** | **OR** |
| --- | --- | --- |
|  | **HNSCC (All Fields)** |  |
|  | **Squamous Cell Carcinoma of the Larynx (All Fields)** |  |
|  | **Laryngeal Squamous Cell Carcinoma (All Fields)** |  |
|  | **Squamous Cell Carcinoma of the Nasal Cavity (All Fields)** |  |
|  | **Oral Tongue Squamous Cell Carcinoma (All Fields)** |  |
|  | **Hypopharyngeal Squamous Cell Carcinoma (All Fields)** |  |
|  | **Oral Squamous Cell Carcinoma (All Fields)** |  |
|  | **Squamous Cell Carcinoma of the Mouth (All Fields)** |  |
|  | **Oropharyngeal Squamous Cell Carcinoma (All Fields)** |  |
| **AND** | | |
| **#2** | **antineoplastic agent (All Fields)** | **OR** |
|  | **cancer chemotherapy (All Fields)** |  |
|  | **cancer combination chemotherapy (All Fields)** |  |
|  | **antitumor agent (All Fields)** |  |
| **AND** | | |
| **#3** | **circRNA (All Fields)** | **OR** |
|  | **Closed Circular RNA (All Fields)** |  |
|  | **Circular RNA (All Fields)** |  |
|  | **Closed RNA (All Fields)** |  |
|  | **Circular Intronic RNA (All Fields)** |  |
|  | **Circular Exonic RNA (All Fields)** |  |
|  | **Exon Intron RNA (All Fields)** |  |
|  | **ecircRNA (All Fields)** |  |
|  | **EIciRNA (All Fields)** |  |

**Search Strategy for Cochrane Library:**

| **#1** | MeSH descriptor: [Squamous Cell Carcinoma of Head and Neck] explode all trees | **OR** |
| --- | --- | --- |
|  | Squamous Cell Carcinoma of the Larynx |  |
|  | Squamous Cell Carcinoma of the Nasal Cavity |  |
|  | Tongue Squamous Cell Carcinoma |  |
|  | Oral Squamous Cell Carcinoma |  |
|  | Hypopharyngeal Squamous Cell Carcinoma |  |
|  | Squamous Cell Carcinoma of the Mouth |  |
|  | Oropharyngeal Squamous Cell Carcinoma |  |
| **AND** | | |
| **#2** | **MeSH descriptor: [Antineoplastic Agents] explode all trees** | **OR** |
|  | **antineoplastic agents** |  |
|  | **cancer chemotherapy** |  |
|  | **antitumor agents** |  |
|  | **anticancer drugs** |  |
|  | **antitumor drugs** |  |
|  | **anticancer drugs** |  |
| **AND** | | |
| **#3** | **MeSH descriptor: [RNA, Circular] explode all trees** | **OR** |
|  | **Circular RNA** |  |
|  | **closed circular RNA** |  |
|  | **Circular Intronic RNA** |  |
|  | **circular exonic RNA** |  |
|  | **ciRNA** |  |
|  | **exon intron circular RNA** |  |
|  | **ecircRNA** |  |
|  | **EIciRNA** |  |
